# Hyaluronan Modulates the Biomechanical Properties of the Cornea

**DOI:** 10.1167/iovs.63.13.6

**Published:** 2022-12-07

**Authors:** Xiao Lin, Taye Mekonnen, Sudhir Verma, Christian Zevallos-Delgado, Manmohan Singh, Salavat R. Aglyamov, Tarsis F. Gesteira, Kirill V. Larin, Vivien J. Coulson-Thomas

**Affiliations:** 1 College of Optometry, University of Houston, Houston, Texas, United States; 2 Department of Biomedical Engineering, University of Houston, Houston, Texas, United States; 3 Department of Zoology, Deen Dayal Upadhyaya College, University of Delhi, Delhi, India; 4 Department of Mechanical Engineering, University of Houston, Houston, Texas, United States

**Keywords:** hyaluronan, alkali burn (AC), cornea, optical coherence elastography (OCE), biomechanical properties, corneal wound healing

## Abstract

**Purpose:**

Hyaluronan (HA) is a major constituent of the extracellular matrix (ECM) that has high viscosity and is essential for maintaining tissue hydration. In the cornea, HA is enriched in the limbal region and is a key component of the limbal epithelial stem cell niche. HA is upregulated after injury participating in the formation of the provisional matrix, and has a key role in regulating the wound healing process. This study investigated whether changes in the distribution of HA before and after injury affects the biomechanical properties of the cornea in vivo.

**Methods:**

Corneas of wild-type (wt) mice and mice lacking enzymes involved in the biosynthesis of HA were analyzed before, immediately after, and 7 and 14 days after a corneal alkali burn (AB). The corneas were evaluated using both a ring light and fluorescein stain by in vivo confocal microscopy, optical coherence elastography (OCE), and immunostaining of corneal whole mounts.

**Results:**

Our results show that wt mice and mice lacking HA synthase (*Has*)*1* and *3* present an increase in corneal stiffness 7 and 14 days after AB without a significant increase in HA expression and absence of scarring at 14 days after AB. In contrast, mice lacking *Has2* present a significant decrease in corneal stiffness, with a significant increase in HA expression and scarring at 14 days after AB.

**Conclusions:**

Our findings show that the mechanical properties of the cornea are significantly modulated by changes in HA distribution following alkali burn.

The cornea is a transparent, avascular, and multilayered tissue that acts as a barrier against various insults from the external environment and, thus, protects the eye.^1^^–^^3^ The cornea is also responsible for most of the refractive power of the eye.^3^ Structurally, the cornea is composed of various layers (i.e. epithelium, Bowman layer, stroma, Descemet's membrane, and endothelium).^1^^–^^4^ The corneal epithelium, the outermost layer of the cornea, plays an important role in maintaining corneal integrity and vision. Because it is prone to damage by various physiochemical and biological insults, its timely replenishment and regeneration are necessary for ocular health and homeostasis achieved by limbal epithelial stem cells (LESCs). The LESCs are a small population of unipotent basal cells that reside in the limbal region within a limbal stem cell niche (LSCN).^5^^–^^8^ The proliferation of LESCs gives rise to progenies that migrate into the cornea and readily replenish the loss of epithelial cells, which is accelerated during wound healing.^8^ Therefore, LESCs are essential for maintaining corneal homeostasis and regenerating a healthy cornea following ocular trauma.^9^^,^^10^ It is estimated that about one-fifth of the population suffers an ocular trauma during their lifetime.^11^ Certain corneal injuries and ocular pathological conditions result in a loss of LESCs, which, in turn, impairs the wound healing process.^10^ The loss of LESCs can lead to LESC deficiency (LSCD), leading to persistent corneal wounds, angiogenesis, conjunctivalization, loss of transparency, and severe ocular pain.^10^ Therefore, understanding how LESCs are supported within the LSCN is critical to limiting their loss after injury and in pathological conditions.^12^^–^^14^

LESCs, like all other cells, are maintained by a fine balance of signaling cues present within the milieu that are primarily dictated by the extracellular matrix (ECM).^12^^,^^15^^–^^19^ Among the main constituents of the ECM, hyaluronan (HA) has been suggested to be essential in regulating LESCs fate in vitro and in vivo*.*^20^^–^^22^ Specifically, conditional inducible knock-out mice lacking hyaluronan synthase 2 (*Has2^Δ/^^ΔCorEpi^* mice), a key enzyme involved in HA biosynthesis within the LSCN, lack LESCs and present characteristics of LSCD.^20^ In addition, *Has2^Δ/^^ΔCorEpi^* mice have increased HA expression throughout the cornea after alkali burn (AB) compared to the wild-type (wt) mice.^20^

The biomechanical properties of the cornea also play an important role in the development, homeostasis, and pathogenesis of the cornea.^16^^,^^17^^,^^23^^–^^26^ For example, the biomechanical properties of the cornea dictate the curvature, stiffness, regularity, and viscoelasticity of the cornea, which together dictate the shape, strength, and transparency of the cornea.^27^^–^^30^ The biomechanical properties of the cornea also have a key role in corneal wound healing.^16^^,^^31^^,^^32^ Recently, studies have demonstrated that the limbal region of the cornea has distinct biomechanical properties when compared to the remaining cornea and that the unique biomechanical profile of the LSCN is necessary to support LESCs.^16^ Changes in the biomechanical properties of the cornea have been shown to affect the LESCs phenotype ex vivo and in vivo*.*^16^^,^^17^^,^^24^^,^^33^^,^^34^ Specifically, LESCs maintain putative stem cell marker expression levels when seeded on relatively soft substrates, whereas they differentiate into corneal epithelial cells when seeded onto stiff substrates.^24^^,^^33^^,^^34^ Additionally, the application of collagenase to the cornea results in a softer limbus and, consequently, supports LESC regeneration in a rabbit AB model.^16^

Because both HA and biomechanics are known to be important players in corneal homeostasis and pathophysiology, we investigated whether changes in HA biosynthesis within the cornea affect the biomechanical properties of the cornea in vivo. Further, we also verified whether changes in the distribution of HA following a chemical injury alter the biomechanical properties of the cornea during the process of wound healing. To test this hypothesis, we compared in vivo biomechanical properties of the cornea after AB in wt mice, combined *Has1* and *Has3 null* mice, and mice lacking HA in the corneal epithelium, namely *Has2^Δ/^^ΔCorEpi^* mice.^20^ For measuring the biomechanical properties of the cornea in vivo, we used quantitative wave-based optical coherence elastography (OCE), which combines an air-coupled ultrasonic transducer to induce elastic wave in corneas and phase-sensitive optical coherence tomography (OCT) for imaging the induced elastic wave.^35^^,^^36^ This allows high-resolution, longitudinal, noninvasive, and in vivo biomechanical imaging of the entire murine cornea in 3D, enabling us to assess the biomechanical properties throughout the cornea. We found that the stiffness of the cornea gradually increases following AB in wt and *Has1* and *Has3 null* mice; however, in contrast, overexpression of HA throughout the cornea in *Has2^Δ/^^ΔCorEpi^* mice after AB leads to a significant decrease in its stiffness. Thus, alterations in the HA content within the cornea after an injury can be used to modify its biomechanical properties and support corneal regeneration.

## Materials and Methods

### Animals

C57BL/6J, combined *Has1* and *Has3 null* (*Has1^−^^/^^−^;Has3^−^^/^^−^*) and *K14-rtTA;TC;Has2^flox/^^flox^* (*Has2*^Δ/ΔCorEpi^) mice were utilized at ages 7 to 8 weeks old. For the *Has2*^Δ/ΔCorEpi^ mice, K14-rtTA (K14; stock number 008099) and tetO-cre (TC; stock number 006224) mice were bred with *Has2* floxed mice (*Has2^flox/^^flox^*) to generate compound K14-rtTA, TC, and *Has2^flox/^^flox^* mice,^37^ namely *Has2*^Δ/ΔCorEpi^ mice, as previously shown.^20^^,^^21^^,^^38^^,^^39^ For *Has2*^Δ/ΔCorEpi^ mice, the *Has2* gene was excised from K14 positive cells upon doxycycline administration (Dox Diet #AD3008S; Custom Animal Diets, LLC, Easton, PA, USA). For such, breeding mice and all weaned mice were maintained on doxycycline chow (Custom Animal Diets, LLC; 200 mg/kg) in lieu of regular chow ad libitum. All mice in the colony were genotyped by PCR using tail DNA. C57BL/6J mice, K14-rtTA, and TC mouse strains were obtained from The Jackson Laboratory (Bar Harbor, ME, USA). For our study, eight *Has1^−^^/^^−^;Has3^−^^/^^−^*, four *Has2*^Δ/ΔCorEpi^ mice, and five wild-type mice were included. All mice were bred and housed in a temperature-controlled facility with an automatic 12-hour light-dark cycle at the Animal Care Facility of the University of Houston. All experimental procedures for handling the mice were previously approved by the Institutional Animal Care and Use Committee at the University of Houston. Animal care and use conformed to the ARVO Statement for the Use of Animals in Ophthalmic and Vision Research.

### Alkali Burn Model

In preparation for the AB, mice were anesthetized by intraperitoneal injection of ketamine hydrochloride (80 mg/kg; #07-890-8598; Vedco Inc., St. Joseph, MO, USA) and xylazine (10 mg/kg; #07-808-1947; Acorn Animal Health, IL, USA). Thereafter, the mice were subjected to AB injuries to the right eye (OD) while the left eye (OS) served as a contralateral uninjured control. AB was induced by instilling 2 µL of 1 N NaOH onto the right corneas of anesthetized mice and left for 1 minute and 20 seconds, as previously described.^36^ Excess tears containing the alkali solution were aspirated using a sterile polyvinyl acetal eye spear (#AX10086; Microsurgical Technology Inc., Redmond, WA, USA), followed by a wash with 5 mL of PBS administered in a continuous dropwise manner. Next, Terramycin ophthalmic ointment (Zoetis Inc., Kalamazoo, MI, USA) was applied to the eyes, and the mice were placed on a warming pad until they were awake. For pain control, mice were provided with Rimadyl tablets (#SMD150-2; Bio-Serv, Flemington, NJ, USA) starting 24 hours prior to the AB. All injuries were carried out at the same time of day to avoid the influence of diurnal changes, and both male and female mice were included in the study.

### Ring Light Assessment of Corneal Smoothness

Corneal smoothness was assessed using a ring light before (baseline), immediately after, and 7 and 14 days after AB, as previously shown.^40^ This was accomplished by analyzing the regularity of a white ring light that was reflected off of the mouse corneas. The regularity of the circle in the reflected image is directly dependent on the smoothness and integrity of the ocular surface.^40^ Epithelial defects and stromal scarring lead to a loss of the ocular surface smoothness, which, consequently, cause distortions to the reflected circular image. The circularity of the reflected ring light was quantified using the shape descriptors measurement plugin in ImageJ 1.52p (National Institutes of Health) and normalized to the wt baseline value. Briefly, we developed a customized script (Supplementary Material) that allows captured pictures to be automatically analyzed and measured by the shape descriptor embedded in ImageJ. The circularity is defined as 4π*area/perimeter^2^. Using this metric, perfectly round reflected circles would have a circularity index of 1 whereas elongated and distorted circles result in decreased values of the circularity. In order to capture images of the reflected ring light, mice were anesthetized, as outlined above, and placed under a ZEISS SteREO Discovery.V12 Modular Stereo Microscope (Carl Zeiss Microscopy LLC, White Plains, NY, USA) and imaged using a white ring light that was cast from the VisiLED Ring Light (#S80-25; Carl Zeiss Microscopy LLC), which was attached to a controller (MC 1100 for VisiLED; Carl Zeiss Microscopy LLC). The ring light was cast onto the cornea using the pupil as a reference to ensure a comparable anatomic location between mice.

### Assessment of Corneal Integrity Using Fluorescein Staining

Corneal epithelial integrity was assessed using fluorescein (GloStrips; Amcon Laboratories Inc., St. Louis, MO, USA) administration before (baseline), immediately after, and 7 and 14 days after AB. For such, mice were anesthetized as outlined above and placed under a ZEISS SteREO Discovery.V12 Modular Stereo Microscope (Carl Zeiss Microscopy LLC). Two µL of a 1 mg/mL fluorescein solution was placed onto the ocular surface, followed by a PBS wash. Images of the ocular surface were acquired immediately under the fluorescence stereomicroscope using the GFP filter.

### In Vivo Confocal Microscopy

Analysis of corneal scarring, haze, and inflammation was carried out using Heidelberg Retinal Tomograph-HRTIII Rostock Cornea Module (HRT-III; Heidelberg Engineering Inc., Heidelberg, Germany), as previously described.^41^ The mice were secured in an adapter designed for rodents, and GenTeal Gel (Novartis Pharmaceuticals Corp., East Hanover, NJ, USA) was applied to the eyeball and between the tip of the HRT-III objective and a TomoCap (Heidelberg Engineering Inc., Franklin, MA, USA) as an immersion fluid. A stack of sequential images (*n* = 40) was captured throughout the cornea as a continuous z-axis scan with 2 µm increments starting from the superficial layer of the epithelium. Images were exported as a sequence of tiff files and analyzed using ImageJ 1.52p. Corneal haze was quantified from the series of images and plotted against corneal depth in order to plot a histogram of the corneal haze throughout the depth of the cornea using in-house scripts. Three representative sequences were obtained per eye per time point. For each experimental point, the data were average to produce a single histogram.

### Elasticity Imaging Using Optical Coherence Elastography 

The OCE system used for imaging the elastic wave propagation mainly comprised of a phase-sensitive spectral-domain OCT (PhS-OCT) system, and noncontact excitation by an air-coupled ultrasonic transducer (ACUS), as described previously.^35^^,^^36^ Specifically, a semi-hemispherical air-coupled piezoelectric transducer with a 10 mm diameter apical circular opening and approximately 20 mm focal distance was used to excite elastic waves. The anesthetized mice were positioned to align excitation at the corneal apex. The generation and amplification of the transducer driving signal, a 3 kHz train of 5 pulses, was achieved by a function generator (DG4162; RIGOL Tech, Beijing, China) and a 55-dB power amplifier (1040L; Electronics & Innovation, Ltd., Rochester, NY, USA), respectively. The M-B mode scanning was performed to track the elastic wave propagation using a PhS-OCT system, which was based on a broadband super-luminescent diode (S480-B-I-20; Superlum Diodes Ltd., Carrigtwohill, Ireland) that had an 840 nm central wavelength and 49 nm bandwidth.^42^^,^^43^ The OCT system had an axial resolution of approximately 9 µm and a displacement sensitivity of 0.28 nm, both in air. Two M-B mode scans were performed along orthogonal planes intersecting at the excitation point, with an A-line acquisition rate of 50 kHz. Each M-mode scan contained 1000 A-lines, and 251 M-mode scans were made over a lateral distance of 3.65 mm and 3.40 mm along the 2 axes. The elastic wave group velocity and the corneal thickness were computed from the OCE data using MATLAB R2021a. The elastic wave group velocity, which indicates the stiffness of the corneas, was computed from the spatio-temporal axial particle velocity map^35^ using the ratio of propagation distance to corresponding time (i.e. the slope in the spatio-temporal image).^44^

### Whole Mount Immunostaining

Following imaging experiments, eyeballs were excised and fixed in 2% buffered paraformaldehyde for 30 minutes. Next, the eyeballs were washed in PBS, the corneas were excised, and four small peripheral incisions were made to enable flat mounting on a slide. Corneas were treated for 30 minutes with 0.2% sodium borohydride (Sigma-Aldrich) and washed in PBS. Corneas were blocked in 10% FBS in PBS containing 0.01 M saponin overnight at 4°C under gentle agitation. Thereafter, cornea whole mounts were incubated overnight with rat biotinylated HA binding protein (HABP-385911; Millipore, Billerica, MA, USA) prepared in block solution at 4°C under gentle agitation and subsequently washed in PBS and further incubated with neutravidin conjugated with Alexa555 prepared in block solution for 8 hours at room temperature. Corneas were finally incubated with DAPI, washed, and mounted in Fluoromount G (SouthernBiotech, Birmingham, AL, USA). Corneas were scanned under an LSM 800 confocal microscope (Carl Zeiss Microscopy LLC) using the tiling mode to obtain z-stacks throughout the entire cornea. The tiles were stitched, and a single orthogonal projection was exported for analysis using ZEN 2.3 microscopy imaging software (Carl Zeiss Microscopy LLC). Total HA content was quantified using ImageJ software Fiji version 2.3.0/1/53q (National Institutes of Health).

### Statistical Analysis

Data analysis was conducted using Microsoft Excel, Datagraph 4.6.1 and GraphPad Prism 9. Descriptive statistics, including corneal ring light parameters, elastic wave velocity, and measured corneal thickness, are presented as mean ± standard deviation (SD) for continuous variables. Multiple comparisons were made between different mice groups and different time points, using ANOVA followed by post hoc tests. A comparison of baseline measurements with follow-up measurements was made within different mouse groups using paired *t*-test. The *p* values < 0.05 were considered statistically significant.

## Results

### Assessment of Corneal Smoothness

**Figure 1.**
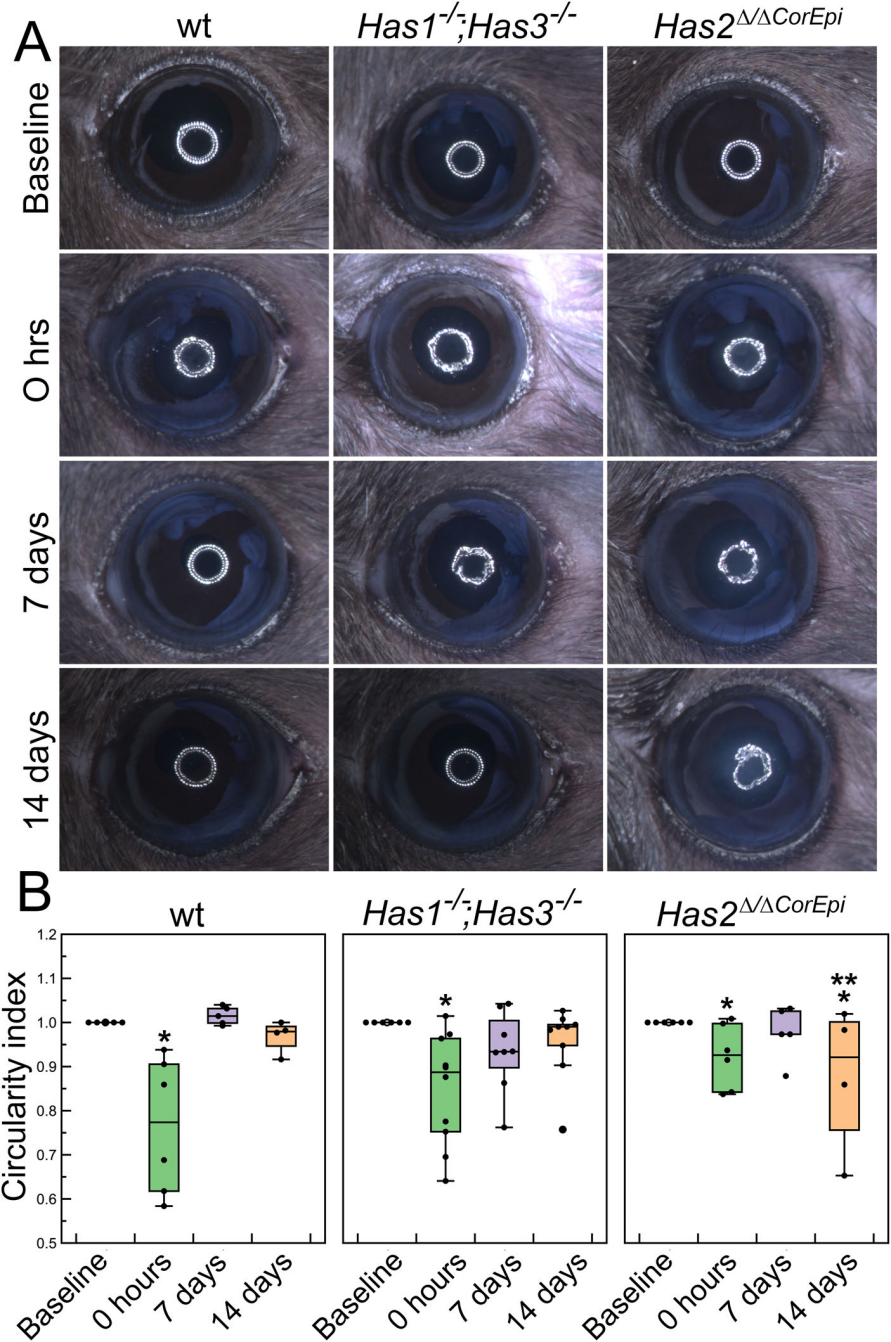
Assessment of corneal smoothness using a ring light projected onto the ocular surface. Corneal smoothness was assessed using a ring light under a stereomicroscope before (baseline - *white*) and immediately after (0 hours), and 7 and 14 days after AB (*black*). (**A**) A white ring light was cast onto the corneas and imaged under a stereo microscope and a representative image presented. (**B**) The circularity of the projected ring light was quantified from an image captured for each mouse as a means to quantify corneal smoothness using ImageJ 1.52p (National Institutes of Health) and data presented as a box plot. * Represents *P* ≤ 0.05 when compared to the baseline of the same genotype, and ** represents *P* ≤ 0.05 when compared to the same time point of the wt mouse. For *Has1^−^^/^^−^; Has3^−^^/^^−^* mice *n* = 8, for *Has2^Δ/^^ΔCorEpi^* mice *n* = 4, and for wild-type mice *n* = 5.

Corneal smoothness was measured using a ring light projected onto the cornea, and the circularity index was calculated using ImageJ software. Epithelial defects and corneal scarring all lead to a loss of corneal smoothness, which, in turn, can be used to assess the corneal wound healing process.^40^ The corneas of all mice included in this study were smooth at the start of the experiment (baseline; Fig. 1). There was a significant loss of corneal smoothness at 0 hours after AB when compared to baseline for each genotype (see Figs. 1A, 1B). The corneal smoothness gradually returned to baseline levels for wt and *Has1^−^^/^^−^;Has3^−^^/^^−^* mice at 7 and 14 days after AB. Additionally, wt and *Has1^−^^/^^−^;Has3^−^^/^^−^* mouse corneas appeared clear at 7 and 14 days after AB. In contrast, for *Has2^Δ^*^/^*^Δ^*^CorEpi^ mice, corneal smoothness did not return to baseline levels at 14 days after AB. In fact, at 14 days after AB, *Has2^Δ^*^/^*^Δ^*^CorEpi^ mice presented a significant loss of corneal smoothness when compared to baseline and when compared to wt and *Has1^−^^/^^−^;Has3^−^^/^^−^* mice 14 days after AB. Additionally, corneas of *Has2^Δ^*^/^*^Δ^*^CorEpi^ mice appear opaque 7 and 14 days after AB.

### Assessment of Corneal Epithelial Barrier Integrity

**Figure 2.**
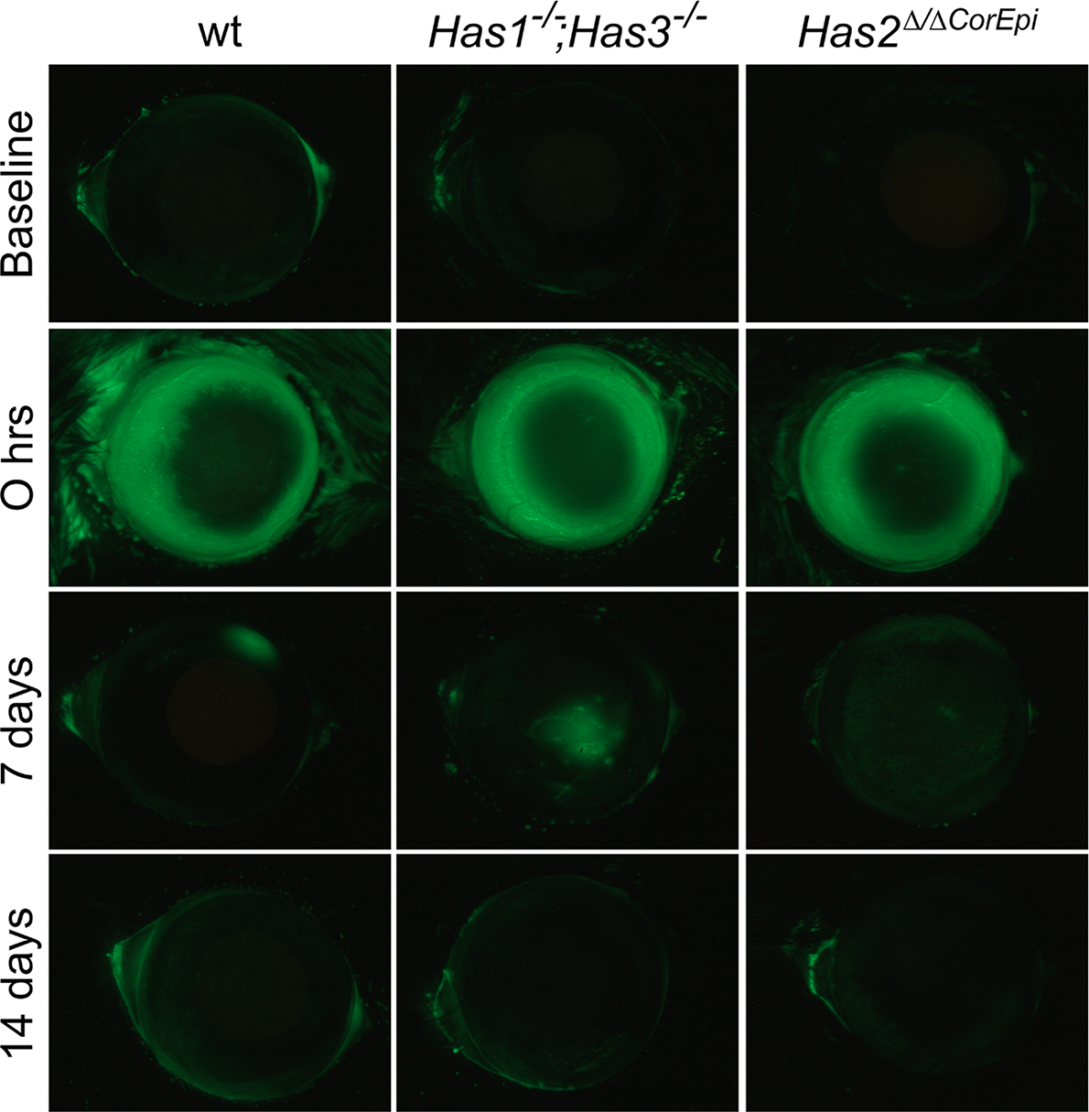
Analysis of wounded area after AB in mice. Images were acquired of the ocular surface of wild-type mice following treatment with fluorescein under a stereomicroscope using the GFP filter before (baseline) and immediately after (0 hours), and 7 and 14 days after AB. For *Has1^−^^/^^−^; Has3^−^^/^^−^* mice *n* = 8, for *Has2^Δ/^^ΔCorEpi^* mice *n* = 4, and for wild-type mice *n* = 5.

Instillation of fluorescein onto the ocular surface was used to assess the injured area, epithelial defects, and loss of barrier function. The corneas of all mice included in this study did not present any corneal fluorescein stain at the start of the experiment (baseline; top row of Fig. 2). At 0 hours after AB, a ring of intense fluorescein stain can be observed around the outer circumference of the cornea, including the conjunctiva, limbal region, and peripheral cornea (see the second row in Fig. 2). Weak fluorescein staining was present throughout the remaining cornea (see Fig. 2). The pattern of fluorescein stain indicates that the NaOH solution that was placed onto the central cornea accumulated around the outer edge of the cornea at the tear meniscus, and therefore caused a more severe burn as a ring surrounding the central cornea (see Fig. 2). At 7 days after AB, a small area of fluorescein stain could be observed on some wt and *Has1^−^^/^^−^;Has3^−^^/^^−^* corneas (see the third row in Fig. 2). At this same time point, most *Has2^Δ/^^ΔCorEpi^* mice presented some punctate fluorescein staining throughout the cornea. At 14 days after AB, no mice presented corneal fluorescein stain (see bottom row of Fig. 2).

### Assessment of Corneal Opacity by In Vivo Confocal Microscopy

**Figure 3.**
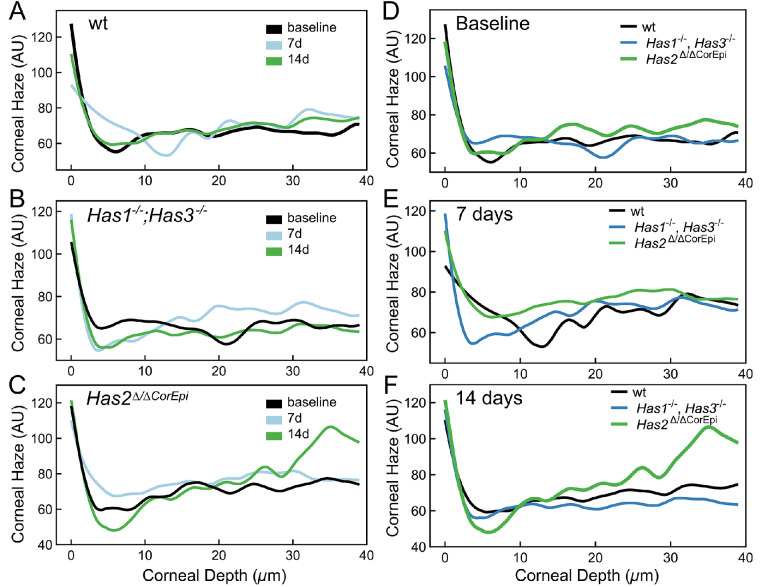
Analysis of corneal haze was analyzed by in vivo confocal microscopy following AB. Analysis of corneal haze and was carried out using Heidelberg Retinal Tomograph-HRTIII with the Rostock Cornea Module. A series of 40 images was collected starting at the outer layer of the corneal epithelium into the corneal stroma as a continuous z-axis at 2 µm increments. The corneal haze was quantified for each image for all mice and data represented as a histogram for (**A**) wt mice (**B**) *Has1^−^^/^^−^;Has3^−^^/^^−^* and (**C**) *Has2^Δ^*^/^*^Δ^*^CorEpi^ mice. The histograms of the mice of different genotypes were also compared at (**D**) baseline, (**E**) 7 days after AB, and (**F**) 14 days after AB. For *Has1^−^^/^^−^; Has3^−^^/^^−^* mice *n* = 8, for *Has2^Δ/^^ΔCorEpi^* mice *n* = 4, and for wild-type mice *n* = 5.

In order to assess corneal haze, corneal opacity was quantified from a sequence of images captured throughout the depth of the corneas starting from the outer layer of the epithelium. There was no significant change in the corneal haze for wt mice at 7 and 14 days after AB when compared to baseline (Fig. 3A). However, for *Has1^−^^/^^−^;Has3^−^^/^^−^* mice, there was an increase in corneal haze throughout the cornea at 14 days after AB (see Fig. 3B). For *Has2^Δ/^^ΔCorEpi^* mice, there was an increase in corneal haze primarily in the posterior cornea 14 days after AB (see Fig. 3C). When comparing the different genotypes, there was no significant difference between the corneal haze throughout the corneas prior to AB (Baseline; see Fig. 3D). However, at 14 days after AB, *Has2^Δ^*^/^*^Δ^*^CorEpi^ mice presented a significant increase in the corneal haze when compared to *Has1^−^^/^^−^;Has3^−^^/^*^−^ and wt mice (see Fig. 3F). This increase in the corneal haze was primarily in the posterior cornea (see Fig. 3F).

### Distribution of HA Throughout the Corneas by Whole-Mount Immunostaining

**Figure 4.**
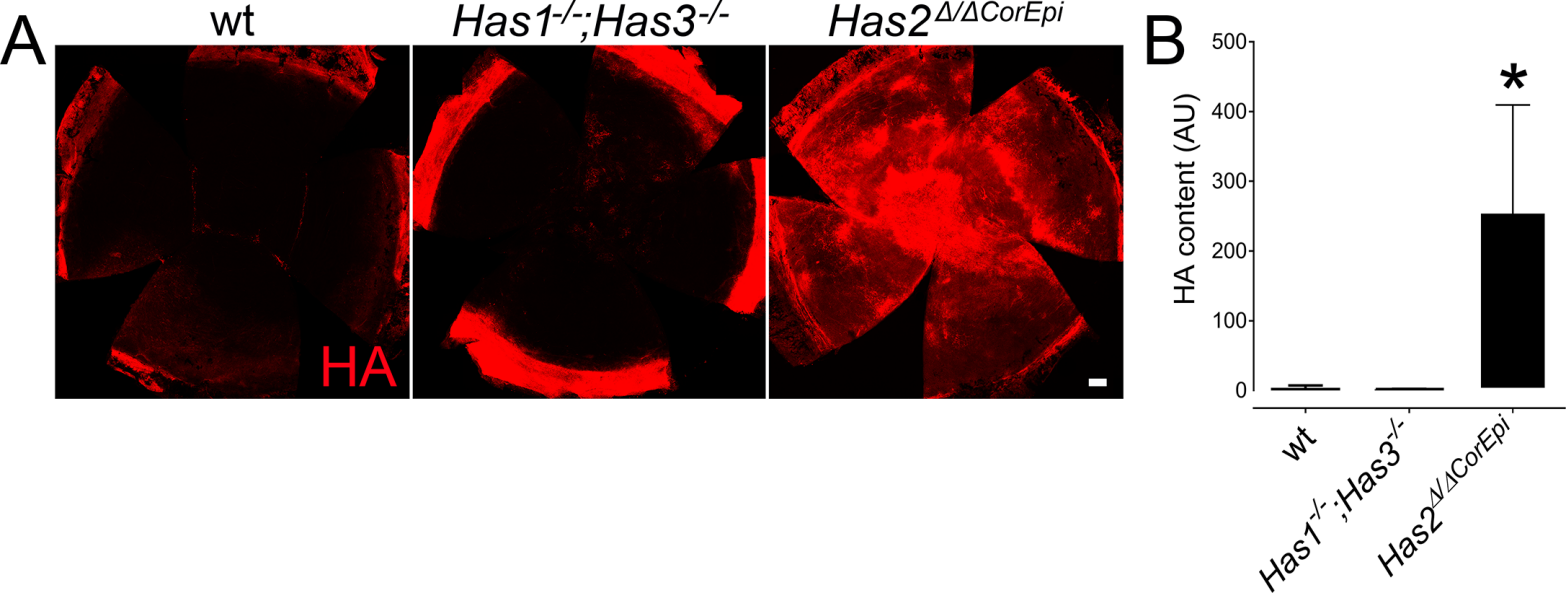
Analysis of HA distribution throughout the corneas 14 days after AB. Corneas were obtained 14 days after AB and processed for HA whole mount staining. Z-stack encompassing the entire cornea were obtained under a confocal microscope using the tiling with the 20 times objective. (**A**) A representative image of HA (*red*) staining in the corneas of wt mice, *Has1^−^^/^^−^;Has3^−^^/^^−^*, and *Has2^Δ^*^/^*^Δ^*^CorEpi^ mice. (**B**) Total number of pixels were quantified within the entire cornea by using a circular demarcation tool in Fiji version 2.3.0/1.53q. * Represents *P* ≤ 0.05. For *Has1^−^^/^^−^; Has3^−^^/^^−^* mice *n* = 5, for *Has2^Δ/^^ΔCorEpi^* mice *n* = 4, and for wild-type mice *n* = 5.

We have previously demonstrated that in the naïve cornea of wt mice, HA is present primarily in the limbal region.^20^ However, naïve *Has1^−^^/^^−^;Has3^−^^/^^–^* mice present a decreased amount of HA within the corneal limbus, whereas naïve *Has2^Δ^*^/^*^Δ^*^CorEpi^ mice present a loss of HA within the corneal limbus when compared to wt mice.^20^ Moreover, our previous work has shown that following corneal injury, *Has2^Δ^*^/^*^Δ^*^CorEpi^ mice upregulate *Has1* and *Has3* expression, and, in turn, express higher levels of HA when compared to wt and *Has1^−^^/^^−^;Has3^−^^/^^−^* mice.^20^ In this study, the distribution of HA throughout the corneas was analyzed by whole mount staining at 14 days after AB (Fig. 4). *Has2^Δ^*^/^*^Δ^*^CorEpi^ mice present significantly higher expression of HA throughout the cornea when compared to wt and *Has1^−^^/^^−^;Has3^−^^/^^−^* mice at 14 days after AB (see Figs. 4A, 4B). HA was distributed throughout the epithelium and stroma of the corneas of *Has2^Δ^*^/^*^Δ^*^CorEpi^ mice (see Fig. 4A).

### Assessment of Corneal Thickness

**Figure 5.**
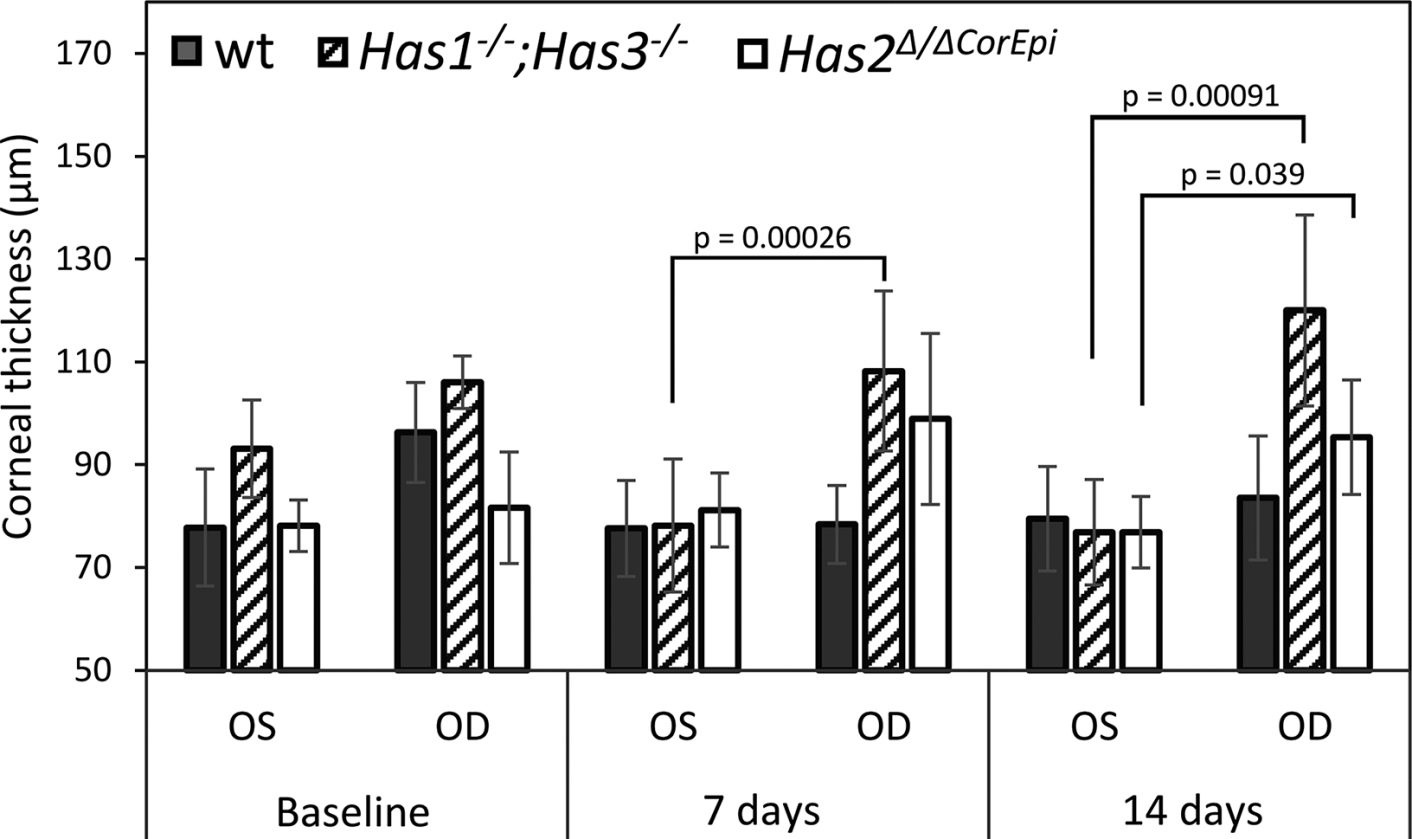
Estimation of the corneal thickness of mice before and after AB. The corneal thickness was quantified in wt mice, *Has1^−^^/^^−^;Has3^−^^/^^−^*, and *Has2^Δ^*^/^*^Δ^*^CorEpi^ mice using OCT B-mode images. For *Has1^−^^/^^−^; Has3^−^^/^^−^* mice *n* = 8, for *Has2^Δ/^^ΔCorEpi^* mice *n* = 4, and for wild-type mice *n* = 5.

Compromised epithelial barrier function and corneal inflammation can both lead to an increase in corneal thickness following corneal injuries.^45^^–^^47^ Given that the thickness of the cornea can also affect its elastic wave speed, we also measured the corneal thickness at baseline, 7 days, and 14 days after AB using OCT images. At baseline, *Has1^−^^/^^−^;Has3^−^^/^^−^* mice presented slightly thicker corneas than wt and *Has2^Δ^*^/^*^Δ^*^CorEpi^ mice; however, this was not statistically significant (*P* > 0.05; Fig. 5). Seven days after AB, *Has1^−^^/^^−^;Has3^−^^/^^−^* mice had significantly thicker corneas when compared to the uninjured eye (*P* = 0.00026). At 14 days after AB, both *Has1^−^^/^^−^;Has3^−^^/^^−^* and *Has2^Δ^*^/^*^Δ^*^CorEpi^ mice had significantly thicker corneas when compared to the uninjured eye (*P* = 0.00091 and *P* = 0.039, respectively; see Fig. 5). Wt mice did not present any significant changes in corneal thickness at any of the experimental time points (see Fig. 5). Representative OCT images have been included in Supplementary Figure S1.

### Biomechanical Properties of the Cornea

**Figure 6.**
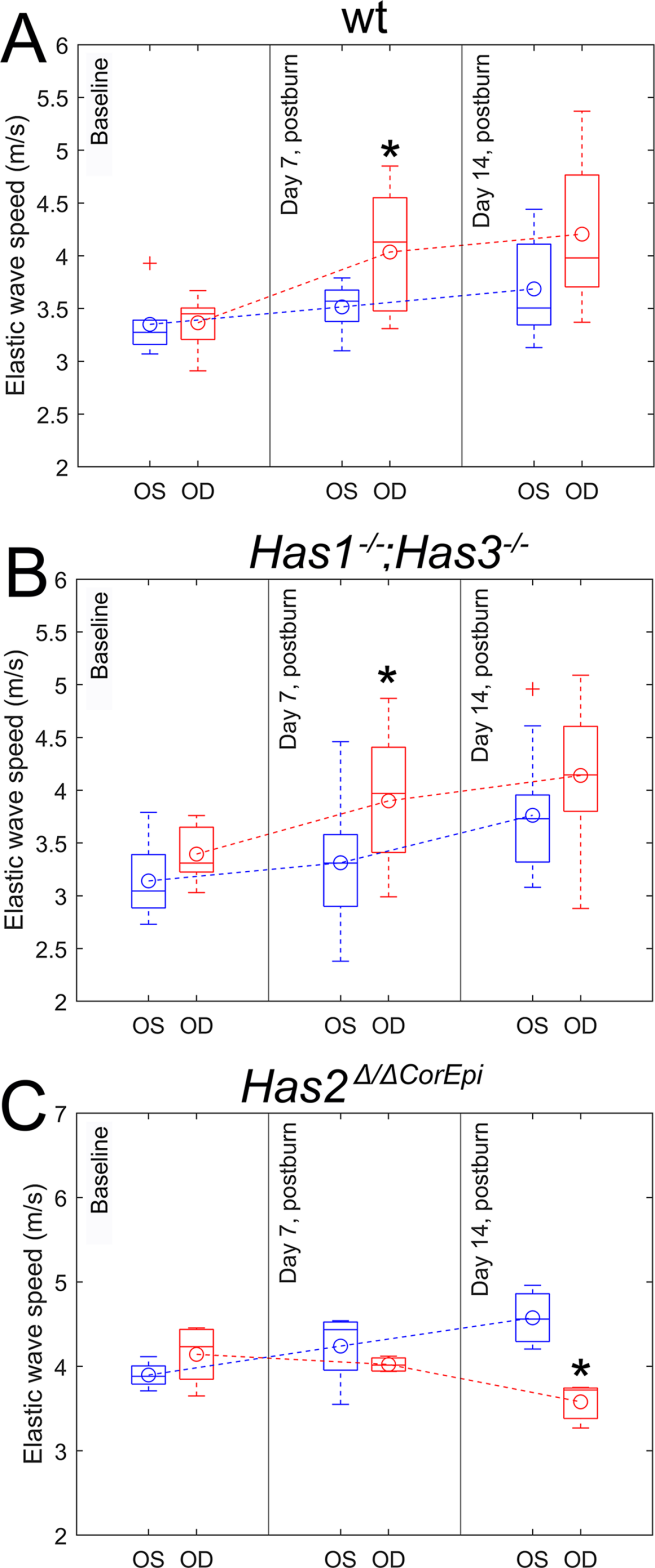
The biomechanical properties of the corneas before and after AB. The mean group velocity was calculated for the corneas of wt mice, *Has1^−^^/^^−^; Has3^−^^/^^−^*, and *Has2^Δ^*^/^*^Δ^*^CorEpi^ mice and data presented as a box-whisker plot. + Represents outliers and * indicate *P* ≤ 0.05 when compared to the uninjured eye. For *Has1^−^^/^^−^; Has3^−^^/^^−^* mice *n* = 8, for *Has2^Δ/^^ΔCorEpi^* mice *n* = 4, and for wild-type mice *n* = 5.

The elastic wave speeds reflecting the mechanical properties of the cornea were quantified from the space-time map of the induced wave propagation using the time-of-flight technique, as previously shown.^35^^,^^48^ When comparing the elastic wave speed of the cornea of the different mice at baseline, both wt and *Has1^−^^/^^−^;Has3^−^^/^^−^* mice had mean elastic wave speeds of approximately ∼3.25 m/s, which were slower than that of *Has2^Δ^*^/^*^Δ^*^CorEpi^ mice (i.e. approximately 4 m/s). As the wave speed in the cornea increases with thickness for a given elasticity, the difference in the wave speed would be higher when corneal thickness (see Fig. 5) is accounted for.^49^^,^^50^ Thus, *Has2^Δ^*^/^*^Δ^*^CorEpi^ mice have a stiffer cornea than wt and *Has1^−^^/^^−^;Has3^−^^/^^−^* mice. Overall, there was a gradual increase in the elastic wave speed through the corneas of contralateral eyes for all mice after AB (Fig. 6). A more pronounced increase in the elastic wave speed was observed through the corneas of the injured eye of wt and *Has1^−^^/^^−^; Has3^−^^/^^−^* mice when compared to the contralateral eye. In stark contrast, there is a gradual and significant reduction in the mean elastic wave speed through the corneas of *Has2^Δ^*^/^*^Δ^*^CorEpi^ mice following AB, from approximately 4.2 m/s at baseline to approximately 4 m/s at 7 days after AB and approximately 3.5 m/s at 14 days (*P* = 0.019) after AB (see Fig. 6). Thus, *Has2^Δ^*^/^*^Δ^*^CorEpi^ mice present a reduction in corneal stiffness following AB when compared to baseline and wt and *Has1^−^^/^^−^;Has3^−^^/^^−^* mice.

## Discussion

The current study aimed to determine whether changes in the composition of the ECM, in particular, changes in the levels of HA, can affect the biomechanical properties of the cornea before and after injury. HA, a major constituent of extracellular matrices, is known to increase the viscosity of tissues, and enables them to withstand compression forces.^51^^,^^52^ In tissues, HA is synthesized by HASs, which are membrane bound glycosyltransferases.^53^^–^^55^ Mammals have 3 highly conserved isoforms, HAS1, 2, and 3, and each HAS is capable of synthesizing HA.^53^^,^^54^ HAS isoforms differ in their spatial and temporal expressions during development, homeostasis, aging, and pathology, however, HAS2 is ubiquitous and the most prevalent isoform in most tissues.^53^^,^^55^^–^^57^ It has been suggested that the molecular weight of the HA produced by the different HASs may vary, with HAS3 primarily synthesizing HA of approximately 100 k to 1000 kDa, whereas HAS1 and HAS2 primarily synthesize HA of approximately 200 k to 2000 kDa.^53^ The loss of Has2 is embryonic lethal, with mice embryos presenting severe cardiovascular abnormalities by E9.5 to 10,^58^ in contrast, double Has1 and Has3 knock-out mice are healthy and viable with a few distinct pathophysiological abnormalities, when compared with wt mice.^59^ Our previous work has demonstrated that naïve mice that lack *Has1* and *Has3* present reduced levels of HA in the cornea when compared to littermate control mice, whereas conditional knock-out mice lacking *Has2* in K14 expressing cells (*Has2^Δ^*^/^*^Δ^*^CorEpi^ mice) present a loss of HA within the epithelial layers of the cornea and limbus.^20^ Curiously, we found that naïve *Has2^Δ^*^/^*^Δ^*^CorEpi^ mice presented increased corneal stiffness, thus we can infer that the loss of HA leads to increased corneal stiffness. Interestingly, when these mice are injured, through a compensatory mechanism, *Has2^Δ^*^/^*^Δ^*^CorEpi^ mice tend to overexpress HA throughout all layers of the cornea when compared to wt and *Has1^−^^/^^−^;Has3^−^^/^^−^* mice.^20^ Thus, together, these mice are a useful tool for studying the effects of altering the HA content in the cornea. In the present study, we longitudinally measured and compared the elastic wave propagation speed through the corneas of wt, *Has1^−^^/^^−^;Has3^−^^/^^−^*, and *Has2^Δ^*^/^*^Δ^*^CorEpi^ mice before and after AB.

HA is a glycosaminoglycan and, as such, is a negatively charged polysaccharide that attracts and retains water in tissues.^60^ HA is a polyanion and has non-Newtonian rheological properties, and in tissues, it plays a role in lubrication, water retention, and shock absorption.^61^^,^^62^^,^^63^ An increase in HA deposition throughout the cornea following injury would, in turn, increase the water content within the cornea.^64^^,^^65^ Previous studies have established that maintaining correct hydration of the corneal stroma is essential for maintaining corneal transparency^66^^–^^68^; thus we can speculate that the increase of HA within the corneas of *Has2^Δ^*^/^*^Δ^*^CorEpi^ mice would contribute to the loss of transparency in the corneas of these mice. Additionally, we hereby demonstrate that the increase in HA throughout the cornea also contributes to a decrease in corneal stiffness. Changes in corneal stiffness and thickness can be induced by altered stromal hydration.^50^^,^^69^^,^^70^ Hydrating the rabbit cornea causes a decrease in corneal stiffness and increased corneal thickness, whereas dehydration of the cornea leads to increased corneal stiffness and reduced corneal thickness, as shown by the OCE and finite-element method technique in situ*.*^50^ Both *Has1^−^^/^^−^;Has3^−^^/^^−^* and *Has2^Δ^*^/^*^Δ^*^CorEpi^ mice, and not wt mice have an unorganized cornea epithelium 2 weeks after AB^20^; however, at 2 weeks, only *Has2^Δ^*^/^*^Δ^*^CorEpi^ mice exhibit decreased corneal stiffness. Therefore, we can infer that the decreased corneal stiffness we observed in the post-burned *Has2^Δ^*^/^*^Δ^*^CorEpi^ mice results from HA-induced hydration and not compromised epithelial barrier function. Interestingly, previous studies have demonstrated that decreasing the corneal stiffness leads to the presence of LESCs within the peripheral and central cornea.^16^ Moreover, substrates with decreased stiffness have been shown to be favorable for maintaining LESCs.^16^^,^^17^^,^^24^^,^^33^^,^^34^ In contrast, increasing corneal and limbal stiffness in vivo leads to an LSCD phenotype in mice.^17^ Thus, based on the literature available to date, corneal stiffness plays an important role in regulating the LESC phenotype. Less stiff environments are more favorable for maintaining LESCs, whereas increasing corneal stiffness can lead to a loss of LESCs. Interestingly, we have previously shown that when *Has2^Δ^*^/^*^Δ^*^CorEpi^ mice upregulate HA expression throughout the cornea, there is a phenotypic switch from corneal epithelial cells to LESCs throughout the entire corneal epithelium.^20^ Therefore, in naïve mice, both HA expression and LESCs are restricted to the limbal region, however, when HA expression is upregulated throughout the entire cornea, in turn, LESCs are present throughout the entire cornea epithelium. Thus, we can speculate that the change in the biomechanical properties of the cornea caused by the increase in the distribution of HA throughout the cornea could contribute to the presence of LESC throughout the corneal epithelium, instead of them being restricted to the limbal region. Similarly, Gouveia and colleagues also demonstrated that decreasing the collagen content in the cornea by collagenase treatment leads to a softer cornea, and, in turn, they also observed the presence of LESCs throughout the entire cornea.^16^ Thus, altering the biomechanical properties of the cornea could be used as a means to promote a more permissive environment for LESCs. Previous studies have documented that the adult limbus is a softer tissue than the cornea.^17^^,^^62^^,^^71^^–^^73^ When measured using mechanical interferometry to estimate the elastic modulus of the corneal and limbal region in humans, the limbus was found to have an elastic modulus of 10 kPa, whereas the central cornea was 17 kPa.^73^ Similarly, when measuring the human corneal and limbal stiffness using Brillouin spectro-microscopy, the limbus presented lower Brillouin shifts when compared to the central cornea, suggesting the limbus is softer compared with the central cornea.^74^ Variations in limbal and corneal stiffness can be influenced by various confounding factors, such as IOP, measurement direction, and subject age.^17^^,^^30^^,^^71^ For instance, in the meridional direction, the central and para-central cornea were found to have the highest Young’s modulus of elasticity.^71^ However, in the circumferential direction, the highest elastic modulus was found at the limbus.^30^^,^^71^ Interestingly, using atomic force microscopy, the murine cornea and limbus showed similar stiffness (1 kPa) at P15, when LESCs are uniformly distributed throughout the cornea; however, at P60, when LESCs are confined to the limbal region, the limbus is softer, with an E value of 2 kPa, compared to the central cornea that has an E value of 7 kPa.^17^

Over the past 2 decades, substantial data have mounted to show that the physiological functions of HA go well beyond its physical contribution to maintaining structural support, H_2_O retention, lubrication, and shock absorption.^51^^,^^52^ HA has been shown to directly regulate various physiological functions, such as cell adhesion, cell proliferation, locomotion, cell cycle, and cell differentiation.^20^^,^^52^^,^^60^^,^^75^ As such, HA has been shown to play a key role in development, homeostasis, inflammation, and pathology.^20^^,^^38^^,^^39^^,^^62^^,^^76^ Many of the physiological functions that have been attributed to HA are believed to be mediated via the direct interaction of HA with various cell surface receptors.^52^ For example, HA has been shown to bind and signal through CD44,^77^ RHAMM,^78^ LYVE-1,^79^ HARE,^80^ and HABP2.^81^ In particular, our group has previously demonstrated that the presence of an HA network within the cornea is necessary for the ingrowth of lymphatic vessels, both during development and in pathological conditions.^39^ Thus, our findings show that HA exerts important physiological effects in the cornea via its direct interaction with cell surface receptors and by modifying the biomechanical properties of the cornea. The high biocompatibility, high water-retention capabilities, viscoelastic properties, and various physiological functions of HA make it a great candidate for use in medicine, and this is especially true in the field of ophthalmology.^82^^,^^83^ For over a decade, HA has been extensively used in various ophthalmic products, including over-the-counter commercially available artificial tears.^83^^,^^84^ Although HA is reported as an inactive ingredient in most eye drops, HA has been reported to exert various beneficial effects when administered to the ocular surface, such as ocular surface hydration, improving the rate of corneal epithelial wound healing, and decreasing ocular surface friction during blinking.^84^^–^^87^ HA has also been explored to reduce contact lens-associated dry eye symptoms by being covalently attached to silicone hydrogel contact lenses, which reduced the rate of dehydration rate and improved contact lens-associated dry eye symptoms.^88^ HA has also been proposed as a great candidate for improving drug delivery to ocular tissues because it can significantly prolong the residence time of drugs^89^^,^^90^ and promote drug absorption.^91^ HA hydrogels that simulate the natural vitreous in terms of transparency, refractive index, and density, are promising vitreous substitutes for treating vitreoretinal diseases.^92^^–^^94^ Our study demonstrates that, additionally, HA-based treatments could be designed to soften the corneal limbal region in LSCD as a means to support LESCs. Stromal collagenase treatment was suggested as an off-label therapeutic strategy to regulate the LESC phenotype by altering the biomechanical properties of the cornea. Our data further support the notion that altering the mechanical properties of the cornea has great potential as a therapy for treating corneal injuries and LSCD. We hereby propose that increasing the HA content within the cornea would not only recreate an LSCN-like niche and support LESCs, but it would also provide more supportive mechanical properties.

This study also revealed a gradual increase in corneal stiffness following AB in *Has1^−^^/^^−^;Has3^−^^/^^−^* and wt mice. Following AB, there is the synthesis of a provisional matrix that favors the wound healing process.^95^ Once the cornea heals, this provisional matrix needs to be gradually modified, enabling the ECM to gradually return to a composition similar to uninjured corneas, supporting corneal homeostasis and transparency.^95^ Previous studies have demonstrated that the provisional matrix tends to have increased stiffness when compared with uninjured corneas.^96^ Murphy et al. used atomic force microscopy to assess the mechanical properties of post-injured corneas.^97^ Their results showed that at 7 days after corneal epithelial debridement wounds and phototherapeutic keratectomy, there was a significant increase in corneal stiffness, which correlated with an increase in stroma haze and the presence of myofibroblasts.^97^ ABs have also been shown to induce corneal stiffening, as demonstrated with human corneas ex vivo by atomic force microscopy^16^ and in mice corneas in vivo by OCE.^36^ The stiffening of the cornea that occurs immediately after AB was attributed to the fact that sodium hydroxide removes proteoglycans from the collagen fibrils, thereby decreasing H_2_O retention,^98^ and the subsequent stiffening has been attributed to the production of the provisional matrix and the wound healing process.^36^^,^^99^ Importantly, it remains to be established whether decreasing or increasing the corneal stiffness via medical interventions could be used as a means of improving corneal wound healing. Curiously, in our study, a gradual increase in corneal stiffness was also observed in the non-injured contralateral eye in all strains of mice, which is consistent with our previous observation in the wt mice.^36^ Previous studies have demonstrated that the contralateral eye often undergoes physiological or pathological changes following insults to the fellow eye.^100^^–^^104^ For example, LESCs were activated to proliferate in the uninjured eye following in vivo corneal scraping of the contralateral eye in the mouse model.^100^ In addition, following AB in one eye, there is a significant increase in the expression of the proinflammatory cytokines substance P and NK-1R in the trigeminal ganglion of both eyes, which may, in turn, trigger changes in epithelial cell proliferation and migration of both eyes.^101^^,^^102^ Sympathetic ophthalmia, a type of uveitis with non-necrotizing inflammatory responses, can occur in both eyes following penetrating injuries, intraocular surgeries, or severe ocular burn of one eye.^103^^,^^104^ Thus, additionally, this study clearly demonstrates that following AB, there is a change in the biomechanical properties of both the injured and uninjured eyes.

Taken together, our study shows that following AB there is a gradual increase in the stiffness of the cornea. However, modifying the ECM by increasing the HA content in the cornea following AB instead leads to a decrease in the stiffness of the cornea. Thus, modifying the composition of the ECM in the cornea after injury by targeting HA could be used as a medical intervention to promote corneal epithelium wound healing.

## Supplementary Material

Supplement 1

Supplement 2
