# Supplementary material for: Hyaluronan Modulates the Biomechanical Properties of the Cornea
Source: Invest Ophthalmol Vis Sci. 2022 Dec 7;63(13):6. doi: 10.1167/iovs.63.13.6 (PMC9733656; doi:10.1167/iovs.63.13.6)
Supplement: Supplement 1 [file iovs-63-13-6_s001.pdf]

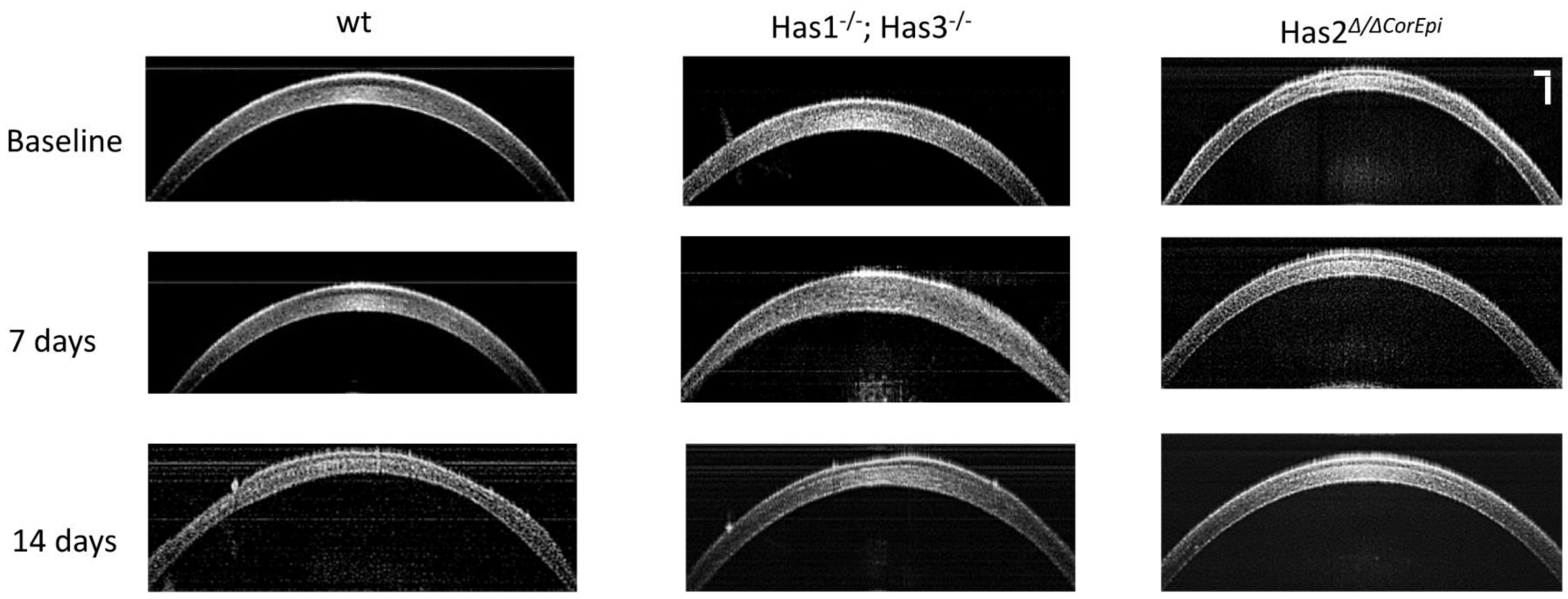

Supplemental material 1: Representative corneal images of wt, *Has1*$^{-/-}$;*Has3*$^{-/-}$ and *Has2*$^{\Delta/\Delta CorEpi}$ mice acquired before (baseline), and 7 and 14 days after AB using the OCT system. The scale bar is 100 µm.
