# Supplementary material for: Hyaluronan Modulates the Biomechanical Properties of the Cornea
Source: Invest Ophthalmol Vis Sci. 2022 Dec 7;63(13):6. doi: 10.1167/iovs.63.13.6 (PMC9733656; doi:10.1167/iovs.63.13.6)
Supplement: Supplement 2 [file iovs-63-13-6_s002.pdf]

```
rep=getDirectory("Choose a folder");
rep2=getDirectory("Creat a folder outside the file folder to save results");
list=getFileList(rep);
for (i=0 ; i<list.length ; i++) {
	nomHA=list[i];
	open(rep+nomHA);
	setOption("BlackBackground", false);
	run("Convert to Mask");
	makeRectangle(582, 398, 726, 864);
	run("Crop");
	run("Close-");
	run("Fill Holes");
	run("Despeckle");
	run("Analyze Particles...", "size=4-17.00 show=Masks display include summarize add");
	roiManager("Select", 0);
	run("Clear Outside");
	a=getTitle();
	saveAs("Tiff", rep2 + a + "processed");
	run("Set Measurements...", "area perimeter shape display label redirect=None 
decimal=3");
	close();
	selectWindow(nomHA);
	close();
	selectWindow("ROI Manager");
	run("Close");
}
selectWindow("Results");
saveAs("Text", rep2+ "roundness");
run("Close");
selectWindow("Summary");
saveAs("Text", rep2+ "particle numbers");
run("Close");
run("Close All");
```

Supplemental material 2: Script used for the quantification of the circularity of the ring light reflected off of the ocular surface.
